# 3D Biofabrication of Thermoplastic Polyurethane (TPU)/Poly-l-lactic Acid (PLLA) Electrospun Nanofibers Containing Maghemite (γ-Fe_2_O_3_) for Tissue Engineering Aortic Heart Valve

**DOI:** 10.3390/polym9110584

**Published:** 2017-11-06

**Authors:** Ehsan Fallahiarezoudar, Mohaddeseh Ahmadipourroudposht, Noordin Mohd Yusof, Ani Idris, Nor Hasrul Akhmal Ngadiman

**Affiliations:** 1 Department of Materials, Manufacturing & Industrial Engineering, Faculty of Mechanical Engineering, Universiti Teknologi Malaysia, 81310 UTM Johor Bahru, Johor, Malaysia; fallahiarezoudar.ehsan@yahoo.com (E.F.); m.ahmadipour87@yahoo.com (M.A.); norhasrul@mail.fkm.utm.my (N.H.A.N.); 2 Department of Bioprocess Engineering, Faculty of Chemical Engineering, Universiti Teknologi Malaysia, 81310 UTM Johor Bahru, Johor, Malaysia; ani@cheme.utm.my

**Keywords:** electrospinning, tissue engineering, heart valve, maghemite nanoparticles, macro-indentation test, response surface methodology

## Abstract

Valvular dysfunction as the prominent reason of heart failure may causes morbidity and mortality around the world. The inability of human body to regenerate the defected heart valves necessitates the development of the artificial prosthesis to be replaced. Besides, the lack of capacity to grow, repair or remodel of an artificial valves and biological difficulty such as infection or inflammation make the development of tissue engineering heart valve (TEHV) concept. This research presented the use of compound of poly-l-lactic acid (PLLA), thermoplastic polyurethane (TPU) and maghemite nanoparticle (γ-Fe_2_O_3_) as the potential biomaterials to develop three-dimensional (3D) aortic heart valve scaffold. Electrospinning was used for fabricating the 3D scaffold. The steepest ascent followed by the response surface methodology was used to optimize the electrospinning parameters involved in terms of elastic modulus. The structural and porosity properties of fabricated scaffold were characterized using FE-SEM and liquid displacement technique, respectively. The 3D scaffold was then seeded with aortic smooth muscle cells (AOSMCs) and biological behavior in terms of cell attachment and proliferation during 34 days of incubation was characterized using MTT (3-(4,5-dimethylthiazol-2-yl)-2,5-diphenyltetrazolium bromide) assay and confocal laser microscopy. Furthermore, the mechanical properties in terms of elastic modulus and stiffness were investigated after cell seeding through macro-indentation test. The analysis indicated the formation of ultrafine quality of nanofibers with diameter distribution of 178 ± 45 nm and 90.72% porosity. In terms of cell proliferation, the results exhibited desirable proliferation (109.32 ± 3.22% compared to the control) of cells over the 3D scaffold in 34 days of incubation. The elastic modulus and stiffness index after cell seeding were founded to be 22.78 ± 2.12 MPa and 1490.9 ± 12 Nmm^2^, respectively. Overall, the fabricated 3D scaffold exhibits desirable structural, biological and mechanical properties and has the potential to be used in vivo.

## 1. Introduction

The four valves in the mammalian heart are responsible for controlling the one-way direction of the blood stream from the heart to body and vice versa [1,2]. For this purpose, the inlet and outlet valves open and close continuously during the cardiac cycles. The malfunction of a valve has resulted in blood circulation disorders and may cause serious heart disease and even death [3]. Currently, when one of the valves malfunctions, the end step of medical choice is to replace it with an artificial one that is state of the art. The drawbacks of artificial valves (either mechanical or biological) such as infection, inflammation, thromboembolic, anticoagulation medication requirement and low durability compel the biomedical engineer to introduce a new concept of tissue engineering heart valve (TEHV) [4]. TEHV is an advanced principle to develop a neo-valvular tissue that can mimic the original tissue characteristics with the capacity to grow, repair and remodel in vivo. Based on tissue engineering (TE) principle, a three-dimensional (3D) scaffold is fabricated using the biomaterials as the initial template for the cells. The shape, structure and mechanical properties of fabricated scaffold should resemble the original aortic heart valve [2,5]. Thus the utilized materials as well as fabrication technique can significantly influence the scaffold properties. 

Electrospinning is a versatile method for fabricating high quality of continuous nanofibers. The nanofibers are accumulated over each other and shape the electrospun mats. The mats obtained have hierarchy structure that is suitable for scaffolding engineered tissue. The 3D scaffold is then seeded with proper source of cells and biological behaviors are investigated. The hierarchy structure of the scaffold facilitated the nutrient supply to the cells (particularly those far away from the surface) as well as waste removal. Later, the cell seeded 3D scaffold is developed in a bioreactor (in vitro) prior to implementation into the body (in vivo). Obtaining a proper structure and morphology of the nanofibers mats via electrospinning requires optimum setting for electrospinning parameters involved as well as solution parameters. Previously, initial evaluation of electrospinning process was performed as reported in several publications [6,7,8,9,10]. In our latest publication [10], the parameters involved such as flow rate, voltage, collector rotating speed and solution parameters such as concentration and ratio of compounds were evaluated to determine the process behavior in terms of mechanical strength. The analysis of results indicated significance and importance level of the investigated parameters. The maximum elastic modulus obtained was roughly 20 ± 1.2 MPa in such a way that elasticity was around 20%, which is not quite desirable for heart valve tissue engineering. It is expected that the scaffold may lose mechanical properties after cell seeding when the degradation rate starts (roughly 10–12 MPa loss is expected during one-month incubation of the scaffold in particular condition) [11,12,13]. However, statistical analysis shows the optimum point is achievable somewhere near the selected range. For this purpose, mathematical techniques such as steepest ascent/descent and response surface methodology are useful to explore this region. These methods enable us to move around the previous experimental range to find the optimum point. Furthermore, a quadratic regression model will be obtained that can be used for further point prediction. The advantage of TE concept compared to the current prosthesis is the potential to mimic the original tissue and no further medication treatment, inflammation and reoperation during the long time is required.

A significant difference between the left and right side of the heart is that the left heart distributes blood to a wider part of the body, naturally achieving a peak pressure six times more than the right side. Subsequently, the mitral and aortic valves on the left side of the heart are exposed to much higher pressure than the pulmonary and tricuspid valves. The wall of the left side is thicker than the right side. Generally, the majority of valvular dysfunctions are related to the left heart’s valves [14,15,16]. The aortic heart valve operates under a dynamic tensile–shear–flexural loading, and tolerates an elastic modulus within 10–15 MPa [11,17]. The heart pumps around 3–5 L blood with velocity around 1.35 ± 0.35 m/s every minute [18]. The suggested value for shear stress value for aortic heart valve is approximately 1–8 Pa, while the peak of this value may increase to a range of 3–150 Pa. In heart valve function, elasticity as well as strength are the two undeniable characteristics [19,20]. 

Several uniaxial tests were performed on both human and animal aortic heart valves to recognize the mechanical behavior. The studies mainly reported an elastic modulus for aortic heart valve in circumferential and radial direction. The reports indicate a much weaker elastic modulus in radial direction compared to circumferential direction. This can be attributed to the direction of collagen fibers that are dispersed circumferentially along the leaflets. In addition, the comparison between human and common animal valves mechanical properties exhibited a much weaker behavior for animals. Balguid and Rubbens [11] reported that the elastic modulus of native human aortic valve to be 15 MPa with ultimate tensile strength of 2.6 MPa and 22% of strain in circumferential direction. In the same report, the elastic modulus in radial direction was measured to be 2 MPa with ultimate tensile strength of 0.4 MPa and strain of 30%. Table 1 lists the related works pertaining to the uniaxial tensile tests of human and animal aortic valve. The average required elastic modulus seems to be approximately 14.5 MPa with at least 22% of elasticity in circumferential direction.

Previously, various types of synthetic/natural biomaterials have been used for scaffolding the heart valve. The synthetic/polymeric based materials must be biocompatible, biodegradable and fulfill the required mechanical properties for the dynamic function of heart valve. Synthetic biodegradable polymers such as polyglycolic acid (PGA) [24,25], polycaprolactone (PCL) [26,27], polylactic acid (PLA) [28,29] , and polyglycerol sebacate (PGS) [30,31] have already been reported for TEHV. Sodian et al. [32] engineered tissue scaffolds using PLA and PGA copolymers where the scaffolds obtained were thicker and less flexible than the individual polymers. Scaffolding PLA polymer has been reported by Armentano et al. [29] and, despite the desired tensile strength, it failed during dynamic mechanism of heart valve leaflets due to its high rigidity (brittleness). Van Lieshout et al. [33] reported the application of PCL in TEHV through the electrospinning technique. The biomechanical behavior was good but the low degradation rate of PCL (more than two years) is a hindrance. Masoumi et al. [30] investigated the scaffolding of PGS in TEHV which demonstrates good biodegradability, stiffness and cell adhesion compared to PGA. The PGS tensile strength tests exhibited nonlinear stress–strain behavior. The average elastic modulus of PGS was within the range of 0.025–1.2 MPa, which was not sufficient for heart valve. The main advantage of a synthetic scaffold is the fact that biomechanics and degradation properties can be chemically controlled according to the requirements. Although no biodegradable polymeric materials have been proven to be a desirable substitute for the native valves, work continues to be promising [1,15,24,25,26,27,28,29].

In our previous study [5], maghemite (γ-Fe_2_O_3_) loaded thermoplastic polyurethane (TPU)/poly-l-lactic acid (PLLA) was used as a novel mixture for fabricating aortic heart valve’s engineered tissue. The presence of maghemite nanoparticles in the nanofibers latter’s surface improved both cell proliferation rate and also the mechanical properties [10,34]. The elastic modulus of the 50:50% (*v/v*) neat TPU/PLLA was improved from 3.24 to 4.76 MPa (roughly 32%) when TPU/PLLA was impregnated with γ-Fe_2_O_3_. However, it is still far away from the required elastic modulus for a heart valve (around 10–15 MPa). Similarly, the cell viability improved from 72.12% to 95.41% during 72 h of incubation. The results indicated 50:50% TPU/PLLA containing 1% γ-Fe_2_O_3_ nanoparticles exhibited overall satisfaction in terms of structural, biological and mechanical properties. The Taguchi analysis was performed to determine the significant parameters influencing the behavior of the selected parameters and the linear regression model was obtained which can only justify the performed experimental range and cannot be generalized for every other process out of that range. In addition, the initial analysis represents half of the curvature (in 3D surface plot) within that range, which means the optimum point is somewhere out of that selected range. Therefore, in this research, the novelty lies in the utilization of initial evaluation and regression model to move from that range to near the optimum point and a second order regression model pertaining to elastic modulus and electrospinning process can be generalized. This was done using the steepest ascent and response surface methodology (RSM). In addition, the cell seeding over the 3D scaffold for 34 days is another novelty of this paper. Besides cell seeding, the new concept is roughness study by AFM techniques for preliminary cell attachment evaluation. Finally, the micro-indentation test was performed after cell seeding to elucidate the elastic modulus loss, which can be attributed to polymer degradation and other factors. 

## 2. Materials and Methods

### 2.1. Materials

Almost all chemical used in this research were purchased and no further purification has been applied on them. Bio-grade PLLA granules (*M*_w_ 70 kDa, glass transition temperature (*T*_g_) 55–60 °C) was purchased from Ingeo™ Biopolymer 4032D (Minnetonka, MN, USA). Bio-grade transparent TPU granules (*M*_w_ 90 kDa, *T_g_* of −50 °C) was purchased from Wenzhou City Sanho Co., Ltd. (Wenzhou, China) The dichloromethane (DCM) and dimethylformamide (DMF), as the solvent for PLLA and TPU, respectively, were purchased from Merck, Co. (Johor Bahru, Malaysia).

The degradability of PLLA and TPU is already verified in terms of changes in the morphology, mass and porosity [5,35] where a 50:50% (TPU/PLLA) scaffold during 24 weeks of incubation in simulated body fluid (SBF), had lose 47.15% of its mass while the morphology showed breakage on the fibers and porosity was increased roughly 15%. The 15 mL of 50:50% TPU/PLLA (*v/v*) (with 6.54 wt % of concentration) solution was prepared by mixing PLLA and TPU solutions using a magnetic stirrer for 5 h, at room temperature (25 °C) to get a homogeneous solution.

In addition the γ-Fe_2_O_3_ nanoparticles were synthesized in laboratory as described in our previous research [36]. Briefly, co-precipitation method [37] was used where ferrous and ferric chloride were combined in stoichiometric in an ammonium hydroxide solution by alkaline co-precipitation for 4 h. Magnetite (Fe_3_O_4_), which is a black and gelatinous precipitate, was obtained. Nitric acid was then used to acidify the precipitate. Isolate the precipitates using magnetic decantation. The solution of ferric nitrate was then used to oxidize it at 100 °C for transforming the solution into γ-Fe_2_O_3_. Citrate anion was then used to coat the γ-Fe_2_O_3_ nanoparticles to prevent agglomeration. The precipitate was washed with acetone and finally dispersed in water resulting in the final stable state γ-Fe_2_O_3_ with a pH of 7. The solution was then freeze dried for 48 h to obtain a soft powder to be dispersed in the TPU/PLLA mixture [6]. 

### 2.2. Response Surface Methodology for Optimizing the Electrospinning Setup

RSM is a collection of statistical and mathematical model to analyze, optimize and model a process. Generally, the RSM is applied after initial analysis (brainstorming) and obtaining the influential parameters on the related process. RSM procedure contains two main steps of steepest ascent/descent and central composite design. The most common model, which is used to define the relationship between vital input parameters and measurable output, is the quadratic regression model, which can be expressed as Equation (1):

(1) $$\hat{Y}=\beta_{0}+\overset{k}{\underset{i=1}{\sum }}\beta_{i}x_{i}+\overset{k}{\underset{i=1}{\sum }}\beta_{ii}x_{i}^{2}+\underset{i<j}{\sum }\sum \beta_{ij}x_{i}x_{j}+\varepsilon$$

where $\hat{Y}$ is the response; β_0_, β*_i_,* β*_ii_* and β*_ij_* are the regression coefficients to be determined; “*k*” represents the number of the factors “*x_i_*”; and “ε” is the statistical error.

In electrospinning method, a high potential electrical field is applied on the emerging solution at the spinneret nozzle tip for transforming the jet into the nanofibers [7,8]. The flow rate of supplied solution was controlled through a syringe pump. The nanofibers were then deposited over the designed 3D collector. The process/system parameters involved such as flow rate (A), voltage (B), percentage of maghemite nanoparticles in the content (C) solution concentration (D) and collector rotating speed (E) were initially analyzed based on 2-level Taguchi experimental design in terms of elastic modulus [9,10] and then were optimized followed by the RSM. Briefly, the initial ANOVA analysis of Taguchi design exhibits the main parameters of voltage (B), percentage of maghemite nanoparticles in the content (C), solution concentration (D) and collector rotating speed (E) and two-way interaction of (BE) significantly effects on the elastic modulus of the nanofibers. The results indicated the parameters setting as flow rate (3 mL/h), voltage (30 kV), percentage of maghemite in the content (3% *w/v*), solution concentration (10 wt %) and collector rotating speed (2000 rpm) were preferred for optimizing the elastic modulus of nanofibers within the applied experimental range where the elastic modulus of 28.49 ± 0.25 MPa was achieved. Based on the Taguchi results the best scaffold was obtained when the temperature was fixed at 25 °C and humidity was less than 10% (controlling the humidity using air-conditioner) [10]. The needle–collector distance was fixed at 10 cm.

Usually, the primary approximation of the optimum operating condition for a process may be far away from the actual optimum. Therefore, in order to follow the optimization procedure the steepest ascent method with respect to the objective need to be applied. The steepest ascent objective is defined as exploring the vicinity of the current experimental region in order to achieve the most efficient direction towards the near optimum area. Using the steepest ascent, the minimum number of experiments would be designed to find the near optimum region. Usually, a linear regression model can fully justify the initial experimental condition. Basically, a perpendicular line to the gradient of the primary linear regression model (which is known as contours line) is the most efficient direction towards the optimum point. The Δ*x_i_* is selected for one significant factor and correspondingly using the coefficients of the regression model; the Δ*x_j_* is computed for the others one. The Δ*x_i_* is given by Equation (2). The experiments were conducted according to the step size along the founded path of steepest ascent until no further increase in elastic modulus was obtained [38,39].

(2) Δxi=βiβjΔxj

where β*_i_* and β*_j_*, are the regression coefficients for two significant factors, and Δ*x_i_* and Δ*x_j_* represent the step size for those factors, respectively.

### 2.3. Elastic Modulus of Electrospun Mats

To investigate the elastic modulus of electrospun mats the uniaxial tensile tests (according to standard testing method ASTM D882) were performed [40]. Tensile strength test was done using universal testing machine (Lloyd, LRX, Singapore,) with a load cell of 0.5 kN at a cross-head speed of 5 mm/min. The electrospun mats consist of a strip of 12.7 mm wide by 76.2 mm in length and 0.3 ± 0.05 mm in thickness. The width of the specimen should not deviate by more than 2% over the length of the electrospun mats between the grips. The thickness of the specimens should not vary by more than 10% over the entire sample. The tests were performed at room temperature (25 °C) in three replicates. The specimens were dry (normal condition after spinning and no further modification was applied prior to testing) during the initial testing.

Elastic modulus (which is a measurement of the materials elasticity) was calculated using Equation (3) [41,42]:

(3) Ee=σ(ε)ε

where the “$E_{e}$” is the elastic modulus, $\sigma_{\left(\varepsilon \right)}$ is the tensile stress and “ε” the tensile strain.

### 2.4. Design of the 3D Aortic Heart Valve’s Template

**Figure 1:**
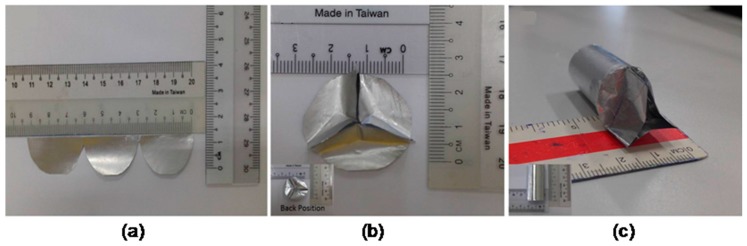
Design procedure of aluminum based 3D template to be used as the collector: (**a**) cutting the aluminum foil into the leaflets shape; (**b**) closing position form of the valve by folding the free-edge length; and (**c**) attaching the leaflets over a cylindrical tube.

To design a 3D template to be used as the collector, an aluminum foil was used. The required dimensions of aortic heart valve were extracted from previous works [43,44,45]. Figure 1 illustrates the procedure of 3D template fabrication. The aluminum foil was cut into the leaflets shape (Figure 1a), formed to the closed position of the valve by folding the free-edge length from the center (Figure 1b) and attached to a foil covered cylindrical tube (Figure 1c) [46]. Normal plastic tape was used to attach the parts together. Later the template was attached as the spinning collector and based on the optimum settings the nanofibers were electrospun over the template. Finally, the aluminum foil was removed from the accumulated nanofibers over that (scaffold). In this design, the 3D scaffold was fabricated easily via one-step.

### 2.5. Structural, Porosity and Surface Roughness Properties

The morphology and diameter distribution of TPU/PLLA-(γ-Fe_2_O_3_) nanofibers were characterized using field emission scanning electron microscopy (FE-SEM, SUPRA 35VP, Oberkochen, Germany) with an accelerating voltage of 10.00 kV. The samples were cut into square shape with dimension 1 × 1 cm^2^. Prior the observation, the samples were coated using a gold sputter coater for 2 min. The diameter distribution of TPU/PLLA-(γ-Fe_2_O_3_) nanofibers was measured using image software (AxioVision Rel 4.8, Oberkochen, Germany). Hundred fibers of each sample were made to determine the average nanofiber diameter. The fibers were selected by the software randomly to prevent the bias. Furthermore, transmission electron microscopy (TEM, JEOL-JSM 6390 L, Chicago, IL, USA) was used for checking the dispersion of maghemite nanoparticles along the latter’s surface of the nanofibers.

The porosity of the 3D scaffold was measured through liquid displacement technique. In this method, a graduated cylindrical bottle was filled with a certain volume of ethanol (*V*_1_). The scaffold was immersed into the bottle for 2 h until it became saturated with ethanol. The volume of ethanol was recorded as (*V*_2_). The electrospun mats was then removed from ethanol and the remained volume of ethanol inside the bottle was recorded as (*V*_3_). The porosity (%) of 3D scaffold was calculated using Equation (4):

(4) $$\mathrm{Porosity}\left(\%\right)=\left(\frac{V_{1}-V_{3}}{V_{2}-V_{3}}\right)\times 100$$

where the “*V*_1_” is the initial volume of ethanol, “*V*_2_” is the volume of ethanol after scaffold immersion, and “*V*_3_” is the volume of ethanol after scaffold removal.

The surface roughness of the electrospun mats was tested using the atomic force microscopy (AFM, 4-sided casted pyramidal tip, Park XE-100, Singapore). For this purpose, the electrospun mats were cut into 0.5 cm × 0.5 cm and the contact mode was utilized. The dimension of the scanning area was 10 µm × 10 µm (the scan angle was fixed at 0.00° and scan rate was at 10 Hz). The quantitative measurement of region statistics was performed, where the “Ra”, “Rq”, and “Rpv” represent the average surface roughness, root mean square surface roughness, and the peak-to-valley roughness of the scanned region, respectively. The higher surface roughness has resulted in better cell attachment.

### 2.6. Cells Viability and Attachment

The aortic smooth muscle cell (AOSMCs) was purchased from Sigma Aldrich^™^ (St. Louis, MO, USA) and cultured based on normal culturing procedure up to passage 7. Briefly, three steps of thawing, plating and sub-culturing of the cells were performed. Initially, a cryopreserved vial of AOSMCs was removed from liquid nitrogen (LN_2_) tank and the cells were thawed by placing the lower half of the vial in water bath at 37 °C for less than 2 min. Next, the cells suspension was pipetted into a centrifuge tube which was already filled by 9 mL of Dulbecco’s modified Eagle’s medium (DMEM) supplemented by 10% (*v/v*) fetal bovine serum (FBS) and 1% (*v/v*) penicillin. The solution was centrifuged at 1100 rpm for 5 min and correspondingly the cells deposited at the bottom of the tube were collected. The supernatant solution was removed and 1 mL of fresh DMEM was added to the tube. The tube was agitated gently to break the pellet and again disperse the cells. The cells suspension was pipetted to a T-75 flask containing 15 mL of DMEM (ratio of medium to surface area at 1:5 mL/cm^2^). The T-75 flask was maintained in a 37 °C, 5% CO_2_ humidified incubator. Every 24 h, the cell confluency was checked and, whenever the cells reached 80% confluency, sub-culturing was applied. To sub-culture the cells, the medium of T-75 flask was aspirated into the waste. Phosphate buffered saline (PBS, 3 mL) was pipetted into the flask and agitated smoothly to wash the monolayer of the cells and remove the remaining DMEM. After washing, the PBS was aspirated and 3 mL of trypsin-inhibitor was poured into the flask (in order to detach the cells from the bottom of flask) and incubated at 37 °C, 5% CO_2_ humidified incubator for 3–5 min. Afterwards, the flask was removed from incubator and the solution (containing trypsin and detached cells) was pipetted to a centrifuge tube that was already filled by 5 mL of DMEM. The solution was centrifuged at 980 rpm for 5 min to pellet the cells. The supernatant solution was aspirated and 2 mL of fresh DMEM was added to the tube. The tube tip was flicked gently to break up the cells clumps and resuspends the cell. Finally, two T-75 flasks containing 15 mL of DMEM were prepared and 1 mL of cells suspension was pipetted into each flask. The flasks were maintained at 37 °C, 5% CO_2_ humidified incubator. This procedure was repeated up to reach passage 7 of the cells. Once the cells were trypsinized one passage was counted. Suitable number of cells (5 × 10^4^ cell/cm^2^) was then seeded over the scaffold loaded into a 50 mL centrifuge tube. The qualitative and quantitative analysis of cells attachment and proliferation were performed using confocal laser microscopy (CLM, Leica-TCS, SP8, Wetzlar, Germany) and MTT assay, respectively, during 15th, 20th and 34th days of incubation. To observe the cells proliferation and attachment, acridine orange/ethidium bromide (AO/EB) staining assay with an excitation maximum at 502 nm and an emission maximum at 525 nm (green) (while in association with RNA the emission maximum shift to 620 nm) was used in such a way that in every mentioned day, the medium was removed and the scaffold was washed with phosphate buffer saline (PBS) twice. The scaffold was then incubated in medium culture supplemented by 80 µL of AO/EB (10 µM) for 20 min. Later, the scaffold was washed with PBS to remove the dye and observed under the CLM within wavelength of 520–610 nm [47].

Furthermore, to measure the cell density, the MTT assay procedure was done. For this purpose, in every mentioned period, the scaffold was removed from the tube and washed twice with PBS and cut longitudinally. The sample was plated in well plate supplemented by 5 mL DMEM and later 80 µL of MTT reagent (5 mg/mL in PBS) was added to the plate and incubated in dark area for 4 h at 37 °C and 5% CO_2_. Then, process was continued by removing the solution from plates and adding DMSO (1 mL) to dissolve the formazan crystals. In addition, similar procedure was done for the tube containing no scaffold (control plate). Then, 100 µL of the solutions were pipetted into a 96- well plate and the cell viability% using Elisa absorbance reader (BioTek, ELx808, Winooski, VT, USA) was measure at 570 nm of wavelength using Equation (5):

(5) $$\mathrm{Cell}\;\mathrm{viability}\left(\%\right)=(\frac{D_{s}}{D_{c}})\times 100$$

where “*D_s_*” is the absorbance value of the scaffold and “*D_c_*” is the absorbance value of control.

### 2.7. Macro-Indentation Test

Macro-indentation flexural test through LF plus materials testing analyzer apparatus (Lloyd Tensile Tester, EZ, Singapore) according to the standard ISO 14577-1 was performed to analyze the deformation and elastic modulus loss percent of scaffold during 15th, 20th and 34th days of incubation after cell seeding. The elastic modulus measurements via macro-indentation bending test was performed easily as compared to very small elongation in compressive or tensile load. To prepare the samples for the test, the scaffold was removed from culture medium at each time point and the leaflets were separated from the root. Three leaflets were cut from commissures and clamped over a round hole with diameter 21 mm with a ball probe (diameter 10 mm). Indentation force of 100 N at quasi-static rate at a cross head speed of 10 mm/min was applied to leaflets until rupture occurred (rupture is defined as a sudden 50% loss in load). To analyze the elastic modulus, the maximum load that can be applied to the leaflets before rupture (N) and maximum extension at break (indentation depth before rupture) need to be translated into stress and strain. The results were analyzed through the applied load versus the depth of indentation by NexygenPlus data analysis software, Unites States. The tests were performed three times (*n* = 3) in two groups of wet and dried scaffolds.

The Hertz theory was used to analyze the force indentation data [48,49]. Equations (6) and (7) represent the procedure for calculating the elastic modulus of scaffold. The relative elastic modulus “*E_r_*” is given by:

(6) $$E_{r}=\sqrt{\frac{9F^{2}}{16R_{e}\delta^{3}}}$$

where “*F*” represents the force applied, “δ” is the depth of indentation, and “*R_e_*” is the contact surface area between ball probe and leaflet. 

The elastic modulus of leaflets (electrospun nanofibers) *E_e_* was calculated by;

(7) $$E_{e}\approx E_{r}\left(1-V_{f}^{2}\right)$$

where *V_f_*, is the Poisson’s ratio of the nanofibers which is assumed as 0.33 [49].

In addition, the bending stiffness of the scaffold was calculated through rupture test. The force was applied to the specimen until the rupture occurred. The linear beam theory (Equation (8)) was used to calculate the stiffness (N·mm^2^).

(8) EI=FL348δmax

where “*EI*” represents the stiffness index, “*F*” is the maximum applied force before rupture, “*L*” is the span length between grids, and “$\delta_{max}$” represents the maximum depth of indentation [50,51].

## 3. Results and Discussions

### 3.1. Optimization of Electrospinning Parameters Involved

**Figure 2:**
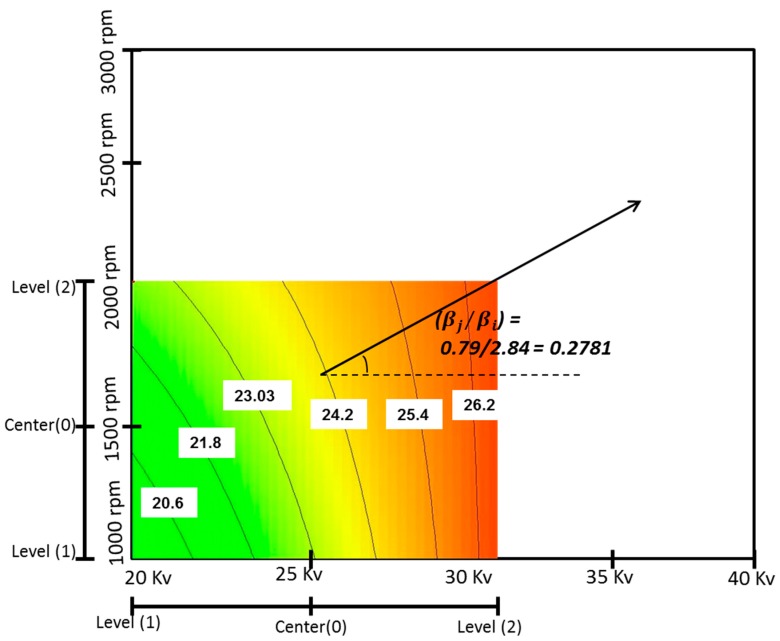
Pathway of steepest ascent method.

The steepest ascent pathway was determined using the ANOVA analysis, the obtained initial linear regression model and the curvilinear in counter plot of response surface. The pathway is a line with slope of ($\beta_{j}/\beta_{i}$) which is perpendicular to the curvilinear where $\beta_{i}$ (2.84) and $\beta_{j}$ (0.79) are the coefficients for factors “*B*” and “*E*”, respectively. Since the counter plot is drawn based on voltage and rotating speed, the coefficient is extracted from these two parameters. Equation (9) shows the calculation for pathway slope. Figure 2 illustrates the steepest ascent pathway with slope of 0.27.

(9) $$Slop=\frac{\beta_{\left(E\right)}}{\beta_{\left(B\right)}}=\frac{0.79}{2.84}=27.81{}^{\circ}$$

Since the flow rate (A) was an insignificant factor on elastic modulus, the variation range was almost very small with value of 0.02 mL/h during the step size determination. The regression model indicated that the elastic modulus increased as a function of voltage increase. Thus, the step size for voltage (B) was defined as increasing by 2 kV to move around the current experimental area. The other parameters step size was calculated based on the increasing rate for voltage. The steps size calculation show a movement from the current optimum design point (flow rate 3 mL/h, voltage 30 kV, percentage of maghemite 3% (*w/v*), concentration 10 wt % and rotating speed 2000 rpm) in current region along the steepest ascent pathway. A 2 kV increase in the voltage resulted in 0.02 mL/h increase in flow rate, 8.9% increase in maghemite content, 11.5% decrease in solution concentration and 55 rpm increase in rotating speed. Table 2 shows the steps size in terms of coded and actual variables.

The electrospun mats were fabricated as per experimental setting and elastic modules were measured through uniaxial tensile tester (LRX 0.5 kN, Lloyd, Singapore). The obtained results are tabulated in Table 3. The results demonstrate an increased in elastic modulus up to third step (from 28.49 to 35.11 MPa). After that, in the fourth and fifth steps the elastic modulus decreased. Thus, it can be concluded that the optimum point was obtainable somewhere around the step three experimental setting.

Near the optimum, the response surface was curved. Therefore, the region needed to be explored more closely by running a series of experiments that support a second order regression model to justify the curvature [38]. For this purpose, central composite design (CCD) was used to design a series of experiments around the obtained optimum point in the steepest ascent pathway (flow rate 3.06 mL/h, voltage 36 kV, maghemite content 3.80%, concentration 6.54 wt % and rotating speed 2165 rpm with elastic modulus 35.11 MPa). The founded point is considered as the center in new experimental range and the vicinity of the region is explored. The star point as (*α* = 1.414) is used in CCD. A small fraction of CCD design with five factors and two center point has resulted in 23 experimental runs. Table 4 depicts the factor levels during CCD design. Furthermore, Table 5 illustrates the CCD design and related responses of elastic modulus.

Significance test of the estimated model as well as the individual model terms and lack-of-fit were performed using the ANOVA analysis. Table 6 tabulates the results obtained refer to these tests. The model’s *p*-value was obtained as <0.0001, which is less than 0.05 and determined a significance level for the model. Likewise, the analysis of the model terms indicated that the main effect of voltage (B) and second order effects of flow rate (A^2^), voltage (B^2^), percentage of maghemite in the content (C^2^) and concentration (D^2^) were significant model terms. The rest of the model terms were insignificant and were removed from the model (except those required to support the hierarchy) to reduce the model noise. The lack-of-fit was insignificant, and its “*p*-value” indicated that the model adequacy due to noise was 43.82%. The main effect of concentration (D) was the most significant factor in elastic modulus. The results also indicated that the (flow rate)^2^, (voltage)^2^, (maghemite)^2^ and (concentration)^2^ provide a secondary contribution to elastic modulus. The value of R-square was 0.9031, which was close to 1 and desirable [9]. This value indicated that the model was able to predict about 90.31% of the variation on the elastic modulus. The difference of Adjusted *R*^2^ value compared to the predicted one was less than 0.2 which shows a reasonable agreement [9]. The range predicted point to design point is compared using the Adeq. Precision. The ratio greater than 4 is desirable [9]; in this case, the value is 13.756.

The following Equations (10) and (11) show the final quadratic regression model in terms of coded and actual variables for elastic modulus:

(10) $$\hat{Y}=35.00-0.57\times \left(A\right)+0.89\times \left(B\right)-0.088\times \left(C\right)+0.44\times \left(D\right)-1.77\times \left(A^{2}\right)-0.98\times \left(B^{2}\right)-1.20\times \left(C^{2}\right)-3.56\times \left(D^{2}\right)$$

(11) $$\begin{array}{l} \mathrm{Elastic}\;\mathrm{Modulus}=42.081+27.024(\mathrm{Flowrate})+17.99(\mathrm{Voltage})+32.36(\mathrm{Maghemite}\%)\\ +110.15(\mathrm{Concentration})-4.420{\left(\mathrm{Flowrate}\right)}^{2}-0.243{\left(\mathrm{Voltage}\right)}^{2}-3.76{\left(\mathrm{Maghemite}\%\right)}^{2}-7.36{\left(\mathrm{Concentration}\right)}^{2} \end{array}$$

**Figure 3:**
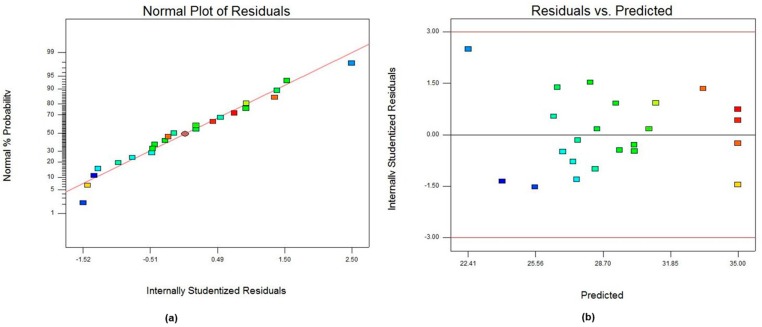
Plots of: (**a**) normal probability of residuals; and (**b**) residual vs. predicted value for CCD analysis.

The diagnostic plots of normal probability and residual versus predicted value plots are depicted in Figure 3. The normal probability plot (Figure 3a) demonstrated a random dispersion of 23 residual points along the regression line which indicated that the errors in the model were distributed normally. In addition, residual versus predicted plot (Figure 3b) exhibited the residual points were scattered randomly without any obvious pattern.

**Figure 4:**
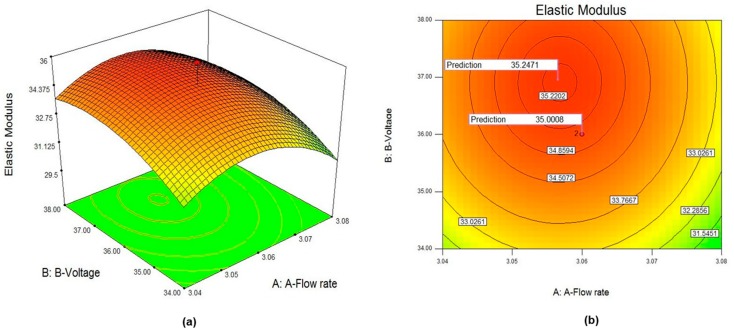
Plots of: (**a**) 3D surface response; and (**b**) counter plot of quadratic model for elastic modulus.

In addition, Figure 4a,b shows the 3D surface and counter plots for elastic modulus response surface in terms of flow rate (A) and voltage (B). The other factors were kept constant at center level. The full curvilinear was visible in both plots, which were in accordance with the quadratic model fitted. The plots exhibited full curvature in experimental range. The counter plot depicted at any particular flow rate; the best surface finished was obtainable when the voltage was somewhere around the middle of the voltage range experimented. This was consistent with the fact that (voltage)^2^ was significant. The results indicated the optimum point was achievable with parameters setting of (flow rate 3.06 mL/h, voltage 36 kV, maghemite content 3.80% (*w/v*), concentration 6.54 wt % and rotating speed 2165 rpm with elastic modulus of 35.24 ± 0.64 MPa). A close exploration around the optimum point revealed a full curvature which exhibited a second order relationship between variables and elastic modulus. The 3D response surface result confirmed the optimum point founded.

### 3.2. 3D Scaffold Biofabrication

**Figure 5:**
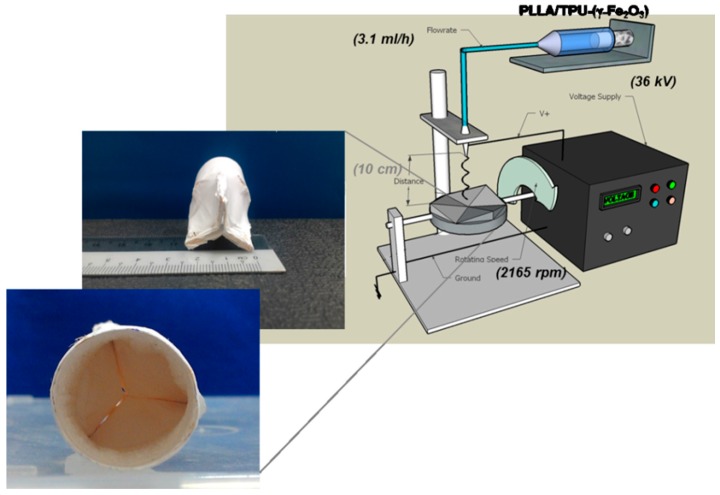
Schematic of electrospinning setup, interior and exterior images of 3D fabricated scaffold.

The designed template was used as the collector to fabricate 3D heart valve scaffold. Fifteen milliliters of the 6.54 wt % of 50:50% (*v/v*) TPU/PLLA solution containing 3.80% (*w/v*) maghemite was prepared. The flow rate, voltage and rotating speed were fixed at 3.06 (approximately considered as 3.1) mL/h, 36 kV and 2165 rpm respectively. Figure 5 illustrates the electrospinning setup and fabricated 3D scaffold using aluminum foil based template. The distance between the needle and collector was fixed at 10 cm.

### 3.3. Structural Properties, Porosity (%) and Surface Roughness

**Figure 6:**
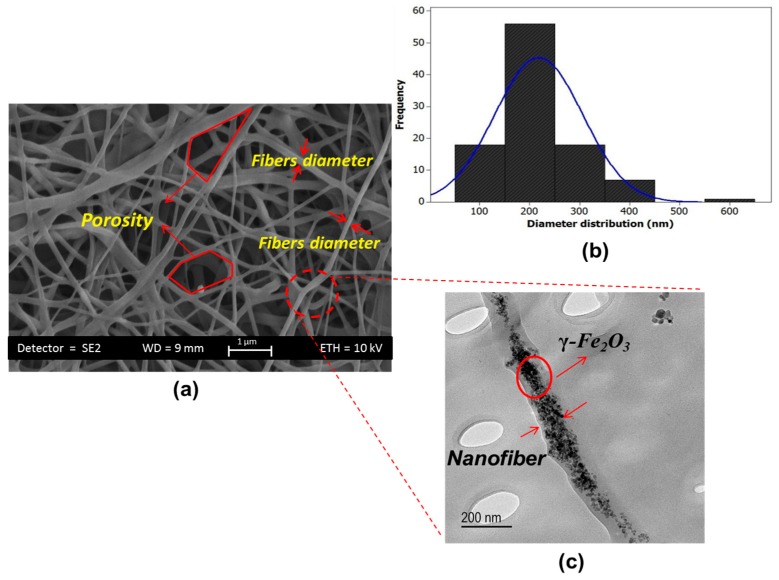
(**a**) FE-SEM image of TPU/PLLA-(γ-Fe_2_O_3_) nanofibers; (**b**) plot of diameter distribution; and (**c**) TEM image.

The FE-SEM image of fabricated 3D scaffold is depicted in Figure 6a exhibiting formation of ultrafine quality nanofibers with no beads. The interconnected pores and hierarchical structure is visible in the image that facilitates the cell attachment and migration. The hierarchical structure provides a channel for supplying the nutrients and removes the waste of the cells that are far away from the scaffold surface. Distribution Plot of nanofibers diameter for the one hundred fibers is depicted in Figure 6b. The majority of fibers were found to have diameter in the range of 178 ± 45 nm in size. Figure 6c illustrates the TEM image of nanofibers where the γ-Fe_2_O_3_ are uniformly scattered along the fibers axis.

Furthermore, it is expected that, when the polymers fully degrade, the maghemite nanoparticles are dispersed in the blood circulation and removed by the spleen or kidney, depending on their size. However, the biocompatibility of maghemite has been verified previously [5,34,52,53].

The porosity (%) was measured by first filling 20 mL of ethanol in graduated bottle. The scaffold was then immersed into the ethanol, resulting in the volume increasing to 20.7 mL after 2 h, the scaffold was removed and the remaining ethanol volume was 13.1 mL. Thus, the 3D scaffold fabricated has porosity of 90.72%. High porosity (high surface-to-volume ratio) is the remarkable advantage that is achieved using electrospinning fabrication technique.

**Figure 7:**
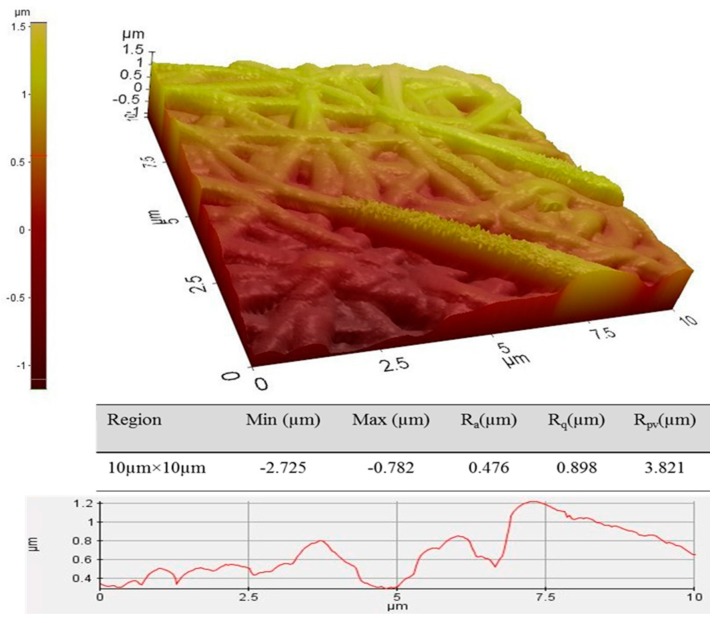
3D surface micrograph of TPU/PLLA-γ-Fe_2_O_3_ surface roughness.

The surface morphology, which is remarkably influential on cell adhesion and proliferation [15,19,41], was investigated using AFM and the 3D surface graphs are depicted in Figure 7. The results indicate that the average of surface roughness is 476 nm. The different colors indicate different heights. It is expected to have a better cell adhesion over the scaffold with higher surface roughness. After total degradation of PLLA and TPU, the maghemite nanoparticles will be dispersed into the blood, circulate along the body and gradually will be filtered by the spleen and kidney as a result of mechanical filtration and are eventually removed by the cells of the phagocyte system.

### 3.4. Aortic Smooth Muscle Cell Viability (%) and Attachment

**Figure 8:**
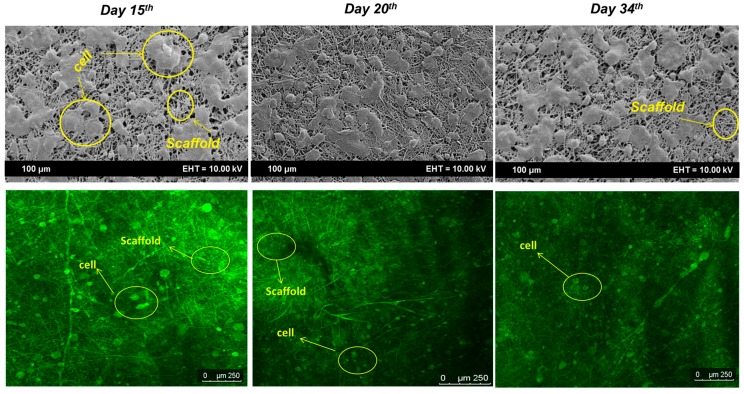
FE-SEM and CLM images of cell attachment on Days 15, 20 and 34 of incubation. The different colors represent the live cells (Green) and scaffold area (Dark).

Confocal laser microscopy (CLM) and FE-SEM images (Figure 8) show the cell growth rate over the scaffold. The green color represents the living cells and the dark area represents the fibers. The images illustrate a desired migration over almost all the scaffold surface and inner layers. The growth rate of cells over the scaffold as a function of incubation time revealed an increasing rate from Day 15 to Day 20 of incubation. The image of Day 20 exhibits the entire surface and inner layers were almost covered with cell. The image on Day 34 represented a similarity with Day 20 of incubation. The images verified the cell adhered very well to the scaffold structure and the nutrients can be supplied even for the cells at the inner layers where the numbers of dead cells were too few to be visible in images. The utilized dyes interact with nucleotides to emit fluorescence.

**Figure 9:**
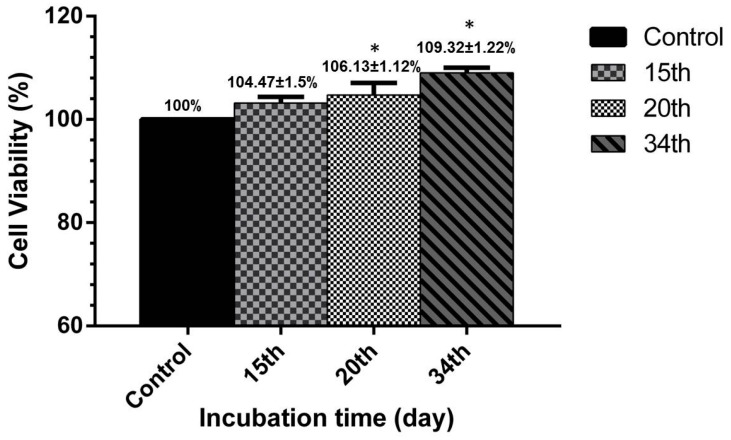
Results of 3D scaffold relative cell viability versus the incubation time (days). Each value is the mean ± SD of all the experiments. * *p* < 0.05 represents significant differences compared to the control analyzed by unpaired *t*-test.

Similar to the results of qualitative analysis, the MTT assay results demonstrated a much desired percent of cell viability during the incubation time. Figure 9 depicts the cell viability percent versus incubation time. It was visible in all the time points that the cell viability over the scaffold was higher than the control (tube without scaffold). Values of 104.47 ± 1.5%, 106.13 ± 1.12%, and 109.32 ± 3.22% represented the viability percent for Days 15, 20 and 34 of incubation, respectively. It was observed that the maghemite nanoparticles in the scaffold matrix led to higher cell viability compared to the control (tube without scaffold) and subsequently formation of extracellular matrix. The same results were obtained in similar works [5,54]. The maghemite particles shaped a microenvironment field in each pore and accelerated the cell growth. The insignificant increased in cell viability within 20 and 34 days of incubation was due to cell reaching the stationary phase of the sigmoid cell life cycle and also the degradation phase of the scaffold. A normal proliferation rate of cells was expected to be achieved over the scaffold matrix as the time goes by.

In addition, the maghemite nanoparticles biocompatibility was tested separately in our previous published work [55]. Briefly, the maghemite nanoparticles were used as the targetable nanoparticles to the folate receptors cancer cells. For confirming the biocompatibility of the γ-Fe_2_O_3_ nanoparticles, normal skin fibroblast cells (HSF-1184) was cultured according to normal procedure and 5 × 10^4^ cell/cm^2^ were cultured in 96-well plate. Maghemite nanoparticles were added to those and incubated for 24 h at 37 °C and 5% CO_2_. The cytotoxicity test (MTT assay) results indicated cell viability >80% which confirms the non-toxicity of maghemite nanoparticles [5,54,55].

### 3.5. Macro-Indentation Test

The macro-indentation test was performed to evaluate the behavior of 3D scaffold after cell seeding and the elastic modulus loss was calculated as a function of incubation time. The test results were divided into two groups of wet and dry scaffolds. The scaffold before cell seeding was used as the control (Day 0). Table 7 summarizes the relevant results to the macro-indentation. The results demonstrated that the incubation time had resulted in loss in mechanical properties such as elastic modulus. As expected, an insignificant difference was obtained between wet and dry scaffold. 

The obtained elastic modulus values of wet scaffold after 15 days of incubation (33.38 ± 1.02 MPa) exhibited around 7.96% loss compared to Day 0 (36.27 ± 0.14 MPa). As the incubation time increased, the elastic modulus decreased with steeper slope where in 20 days of incubation elastic modulus decreased by around 14.76% (28.45 ± 2.03 MPa) compared to Day 15. This was attributed to the higher degradation rate in this time interval. The elastic modulus for Day 34 of incubation (22.78 ± 2.12 MPa) indicated a 37.19% loss compared to Day 0. Even though a significant loss percent was obtained, the elastic modulus was approximately 50% more than what is required for aortic heart valve scaffold. 

**Figure 10:**
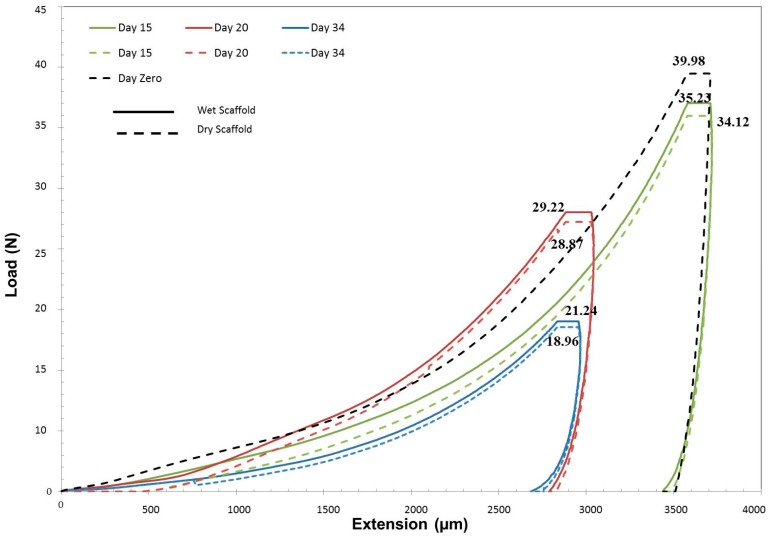
Load vs. extension curve for 3D scaffold after 15, 20 and 34 days of cell seeding.

In a similar trend, the elastic modulus results of dry scaffold had an approximately 40.55% loss on Day 34 compared to Day 0. The measured elastic modulus for 15, 20 and 34 days of incubation for dry scaffold was 32.34 ± 1.18, 28.11 ± 1.32 and 21.56 ± 2.16 MPa, respectively. Figure 10 illustrates the load (N) versus extension (µm) results of macro-indentation test. The loss of elastic modulus is in similar trend with the degradation rate of polymers. Even though the found optimum elastic modulus may be much higher than the required value, the degradation may affect the performance of developing tissue before the complete development in lower modulus range.

## 4. Conclusions

This study has succeeded in demonstrating that nanofiber based scaffold fabrication using (γ-Fe_2_O_3_) nanoparticles loaded thermoplastic polyurethane/poly-l-lactic acid (TPU/PLLA) has the potential to be a suitable choice for tissue engineering aortic heart valve. Steepest ascent followed by the RSM was performed as the systematic method to optimize the elastic modulus of electrospun mats where the value was improved approximately 24% (from 28.49 to 35.11 MPa). Even though the found optimum elastic modulus may be much higher than the required value, the degradation may affect the performance of developing tissue before the complete development in lower modulus range. Ultrafine quality nanofibers with diameter distribution of 178 ± 45 nm were achieved. The 3D scaffold was fabricated easily with proper structure and remarkable porosity (90.72%), which is desirable for cell seeding. The relative cell viability represented higher rate of proliferation over the 3D scaffold compared to the control in all investigated days. Macro-indentation study on the mechanical behavior of seeded scaffold exhibited 7.96%, 21.56%, and 37.19% loss in elastic modulus for Days 15, 20, and 34 of incubation, respectively, compared to Day 0.

## Figures and Tables

**Table 1:** Uniaxial mechanical properties of human and animal aortic valve.

Group	Elastic Modulus (MPa)		Tensile Strength (MPa)		Tensile Strain (%)		Reference
	Circum.	Radial	Circum.	Radial	Circum.	Radial	
Human	15	2	2.6	0.4	22	30	[11]
	14.55 ± 3.7	1.57 ± 0.1	2.3 ± 0.17	0.2 ± 0.21	6.8 ± 1.9	6.9 ± 1.9	[21]
	13.1	7.5	1.7	0.31	9	13	[12]
Animals	7.78 ± 1.7	1.28 ± 0.3	0.73 ± 0.1	0.1 ± 0.01	16.8 ± 6.5	11.6 ± 3.1	[21]
	9.26	2.28	1.2	0.32	39	51	[22]
	6.6	1.33	0.68	0.06	8	N/A	[23]

**Table 2:** Steepest ascent pathway design in terms of coded and actual variable.

Run	Group	Coded Variables					Actual Variables				
		*X*_1_	*X*_2_	*X*_3_	*X*_4_	*X*_5_	ξ_1_	ξ_2_	ξ_3_	ξ_4_	ξ_5_
	Origin Δ = Step Size	2	2	2	1	2	3	30	3%	10%	2000
		0.042	0.4	0.26	−0.46	0.11	0.02	2	8.9%	−11.5%	55
1	Origin + 1Δ	1.042	1.4	1.26	−1.46	1.11	3.02	32	3.26%	8.85%	2055
2	Origin + 2Δ	1.084	1.8	1.53	−1.92	1.22	3.04	34	3.53%	7.70%	2110
3	Origin + 3Δ	1.126	2.2	1.80	−2.38	1.33	3.06	36	3.80%	6.54%	2165
4	Origin + 4Δ	1.168	2.6	2.06	−2.84	1.44	3.08	38	4.06%	5.40%	2220
5	Origin + 5Δ	1.210	3.0	2.33	−3.30	1.55	3.10	40	4.33%	4.25%	2275

**Table 3:** Responses of tensile strength, tensile strain and elastic modulus.

Run	Group	Response		
		Tensile Stress (MPa)	Tensile Strain%	Elastic Modulus (MPa)
	Origin	6.86	24%	28.49
1	Origin + 1Δ	7.23	22.8%	31.71
2	Origin + 2Δ	7.35	21.8%	33.61
3	Origin + 3Δ	7.76	22.1%	35.11
4	Origin + 4Δ	6.10	20.0%	30.38
5	Origin + 5Δ	4.10	15.0%	27.30

**Table 4:** Factors and levels for CCD design.

No.	Factors	Levels				
		(α = −1.414)	(−1)	Center (0)	(+1)	(α = 1.414)
1	A-Flow rate (mL/h)	3.031	3.04	3.06	3.08	3.088
2	B-Voltage (kV)	33.17	34	36	38	38.82
3	C-Maghemite (%)	3.42	3.53	3.80	4.06	4.17
4	D-Concentration (wt %)	4.91	5.40	6.54	7.70	8.16
5	E-Rotating speed (rpm)	2087	2110	2165	2220	2242

**Table 5:** Small fraction of CCD layout and responses of elastic modulus (MPa).

Run	Experimental Points	Factors					Response
		*A*	*B*	*C*	*D*	*E*	*E_e_* (MPa)
1	Factorial Points	1	1	−1	1	−1	30.65
2		1	−1	1	1	−1	28.51
3		−1	1	1	−1	1	28.64
4		1	1	1	−1	−1	26.63
5		1	1	−1	−1	1	26.17
6		1	−1	−1	1	1	28.28
7		−1	−1	1	1	1	27.32
8		−1	1	−1	1	1	28.91
9		1	−1	1	−1	1	23.92
10		−1	1	1	1	−1	32.10
11		−1	−1	−1	−1	−1	26.26
12	Axial Points	−1.4.14	0	0	0	0	29.73
13		1.414	0	0	0	0	29.52
14		0	−1.414	0	0	0	29.89
15		0	1.414	0	0	0	34.63
16		0	0	−1.414	0	0	32.17
17		0	0	1.414	0	0	31.01
18		0	0	0	−1.414	0	24.65
19		0	0	0	1.414	0	22.73
20		0	0	0	0	−1.414	35.54
21		0	0	0	0	1.414	33.14
22	Center points	0	0	0	0	0	35.95
23		0	0	0	0	0	34.68

**Table 6:** ANOVA table for quadratic model after model reduction.

Source	Sum of Squares	df	Mean Square	*F*-Value	Prob. > F	Status
Model	279.672	8	34.95902	16.32379	<0.0001	Significant
A-Flow rate	5.55177	1	5.551778	2.592352	0.1297	-
B-Voltage	13.6666	1	13.66606	6.381241	0.0242	Significant
C-Maghemite%	0.13369	1	0.133696	0.062428	0.8063	-
D-Concentration	3.29399	1	3.293995	1.5381	0.2353	-
A^2	56.6745	1	56.67451	26.46364	0.0001	Significant
B^2	17.2337	1	17.23371	8.047122	0.0132	Significant
C^2	26.2403	1	26.2403	12.25267	0.0035	Significant
D^2	229.459	1	229.4592	107.1439	<0.0001	Significant
Residual	29.9823	14	2.141599	-	-	-
Lack of Fit	29.1866	13	2.245129	2.821554	0.4382	not significant
Pure Error	0.7957	1	0.795707	-	-	-
Cor. Total	309.654	22	-	-	-	-
Std. Dev.	1.46342	R-Squared		0.9031	**-**	**-**
Mean	29.2768	Adj R-Squared		0.8478	**-**	**-**
C.V. %	4.99855	Pred R-Squared		0.6448	**-**	**-**
PRESS	109.960	Adeq Precision		13.756	**-**	**-**

**Table 7:** Macro-indentation test result for 3D optimum scaffold after cell seeding.

Day	Wet Scaffold				Dry Scaffold			
	Load	Extension	Elastic Modulus	Stiffness	Load	Extension	Elastic Modulus	Stiffness
	(N)	(µm)	(MPa)	(Nmm^2^)	(N)	(µm)	(MPa)	(Nmm^2^)
**0**	N/A	N/A	N/A	N/A	39.98 ± 2.1	3510 ± 32	36.27 ± 0.14	2197.5 ± 32
**15**	35.23 ± 1.43	3422 ± 22	33.38 ± 1.02	1986.2 ± 26	34.12 ± 1.4	3467 ± 21	32.34 ± 1.18	1898.6 ± 41
**20**	29.22 ± 2.32	2840 ± 34	28.45 ± 2.03	1985.0 ± 17	28.87 ± 1.2	2870 ± 34	28.11 ± 1.32	1940.7 ± 22
**34**	21.24 ± 1.54	2720 ± 26	22.78 ± 2.12	1490.9 ± 14	18.96 ± 1.8	2798 ± 27	21.56 ± 2.16	1307.3 ± 32
